# Bidirectional Relationship Between Nutrition and Mental Health and Its Impact on the Health of Canadian Immigrants: An Integrative Review

**DOI:** 10.3390/healthcare13080850

**Published:** 2025-04-08

**Authors:** Naika Dubois, Isabelle Giroux

**Affiliations:** 1 Interdisciplinary School of Health Sciences, Faculty of Health Sciences, University of Ottawa, Ottawa, ON K1N 6N5, Canada; 2 School of Nutrition Sciences, Faculty of Health Sciences, University of Ottawa, Ottawa, ON K1N 6N5, Canada; 3 Institut du Savoir Montfort, Ottawa, ON K1K 0M9, Canada

**Keywords:** nutrition, health, mental health, immigrants, Canada, food, diet, food insecurity, obesity

## Abstract

**Background**: Immigration is a key factor contributing to population growth in Canada, a trend that is expected to continue. Immigrants generally arrive with better health than the Canadian-born population, but this advantage often diminishes over time, partially due to dietary acculturation. Emerging evidence points to a bidirectional link between nutrition and mental health. **Objective**: To explore the bidirectional relationship between nutrition and mental health and its impact on the health of Canadian immigrants, with a specific focus on immigrants’ mental health and the healthy immigrant effect. **Methods**: For this integrative review, two comprehensive literature searches were conducted in the databases MEDLINE, CINAHL, Embase, PsycINFO, Scopus, and Web of Science from inception to July 2024. The review adhered to Whittemore and Knafl’s integrative methodology, with the Mixed Methods Assessment Tool used to assess the quality of the studies. **Results**: A total of 42 and 34 scientific articles were included from the first and second literature searches, respectively. Four main themes emerged from the literature: (1) food insecurity and mental health, (2) obesity and mental health, (3) diet quality and mental health, and (4) the gut microbiome and mental health. These themes were explored in the context of Canadian immigrants’ health. **Conclusions**: The health of immigrants to Canada is likely shaped by complex, bidirectional interactions among various determinants of health, influencing both physical and mental well-being. As newcomers are expected to form an increasing proportion of the Canadian population, further research is needed to understand how the interaction between nutrition and mental health can help promote and safeguard the health of Canadian immigrants.

## 1. Introduction

Canada has a long-standing and diverse history of immigration. As the Canadian population continues to age and fertility rates remain below replacement levels, immigration has become the primary contributor to population growth, a trend expected to persist in the coming decades [1]. The existing literature on the healthy immigrant effect (HIE) indicates that immigrants tend to be healthier than the Canadian-born population at the time of their arrival [2]. However, this health advantage tends to diminish over time [3,4,5]. Migration is increasingly recognized as a determinant of mental health, with challenges related to cultural adaptation and integration contributing to a decline in immigrants’ psychological well-being [3,6]. This decline is thought to be partly driven by changes in diet associated with the process of acculturation [3,6].

Studies also show that immigrants’ risk factors for cardiovascular disease (CVD) and chronic conditions, such as diabetes, increase the longer they live in Canada, even after accounting for various confounding factors [7,8]. Moreover, unhealthy dietary patterns have been associated with exacerbations in anxiety, depression, and other neuropsychiatric conditions [9]. A growing body of evidence suggests that mental health and eating behaviors are interconnected through bidirectional relationships [9,10].

A deeper understanding of the two-way connection between nutrition and mental health is essential in addressing the well-being of Canadian immigrants. Such insights are critical for informing policies and practices designed to support the health of newcomers and prevent health declines after migration. 

In this regard, this review aimed to synthesize the current understanding of the bidirectional relationship between nutrition and mental health and explore how this interaction may influence the nutritional and mental health outcomes of immigrants in Canada. 

## 2. Materials and Methods

An integrative review methodology was used to synthesize the literature in this study. This approach facilitates the creation of a thorough summary of research on a broad topic, offering deeper insights into the available evidence [11,12]. The integrative review allows for the inclusion of various study designs, resulting in a comprehensive overview that deepens the understanding of the phenomenon under investigation [12,13]. The review process adhered to the five steps outlined by Whittemore and Knafl (2005): (1) problem identification, by clearly defining the research problem and purpose; (2) literature search, using a well-defined search strategy by utilizing databases and additional methods to ensure comprehensive coverage of relevant literature; (3) selecting and evaluating data, assessing studies based on criteria specific to different methodologies; (4) data analysis, coding and categorizing information to derive key themes and conclusions while integrating diverse study designs; and (5) presentation, effectively summarizing findings using visual tools such as tables to facilitate comparisons across studies [12].

### 2.1. Search Strategy

Two comprehensive literature searches were conducted for this integrative review.

Part 1: The first search addressed the research question: “What is the current state of knowledge in the literature regarding the bidirectional relationship between nutrition and mental health”?

Part 2: The second literature search explored the themes identified in the initial one, focusing on their relevance to Canadian immigrants. It also included an overview of migrants’ mental health and the HIE.

The databases utilized for both searches were MEDLINE, CINAHL, Embase, PsycINFO, Scopus, and Web of Science, covering publications from inception to July 2024. Hand searches for bibliographic citations on relevant material were also included. For both searches, the inclusion criteria consisted of peer-reviewed scientific articles available in full text, published in English or French due to the authors’ language proficiency, and without restrictions on publication year. The exclusion criteria encompassed commentaries, editorials, grey literature, unpublished manuscripts, and dissertations. Additionally, articles that did not provide relevant information on the research topic were excluded. Several keywords and medical subject headings were used through different combinations to capture as many relevant results as possible. The first literature search included the terms: “nutrition”, “diet”, “food”, “mental health”, “depression”, “anxiety”, “mental illness”, “bidirectional relationship”, along with relevant synonyms and MeSH terms for each database. The second search expanded to include “food insecurity”, “obesity”, “diet quality”, “gut microbiome”, “mental health”, “depression”, “anxiety”, “mental illness”, “immigrants”, “refugees”, “migrants”, “Canada”, “healthy immigrant effect”, with corresponding synonyms and MeSH terms. Keywords were combined using the Boolean operators “AND” and “OR”. Truncations and wild cards were also applied to broaden the number of results. The scope of the review was intentionally wide in order to provide a more comprehensive grasp of the subjects. Results were imported into Covidence software (www.covidence.org) for duplicate removal and initial screening of titles and abstracts. Selected articles were then transferred to Zotero software v.6.0.36 for full-text screening.

### 2.2. Data Analysis and Quality Assessment 

A thematic analysis approach was employed for data analysis, following the method outlined by Popenoe et al. (2021) and Dwyer (2020) [14,15]. This inductive method emphasizes the identification of patterns and answers within the data without relying on preconceived theories or analytical frameworks [14,15]. In accordance with this approach, data extraction tables were developed to summarize the characteristics of all selected studies. Relevant findings from each study, referred to as data units, were recorded in a data analysis table. These data units were then organized into summaries, codes, subcategories, and categories, as described by Popenoe et al. (2021) [14]. This systematic process facilitated the identification of overarching themes emerging from the initial literature search.

The quality of the included studies was assessed by both researchers through consensus, using the Mixed Methods Appraisal Tool (MMAT). This tool was selected for its ability to comprehensively evaluate quantitative, qualitative, and mixed-methods studies, making it particularly appropriate for integrative reviews [16]. The MMAT employs five key criteria and has demonstrated reliability, user-friendliness, and efficiency [17].

## 3. Results

### 3.1. Characteristics of Included Studies

**Figure 1:**
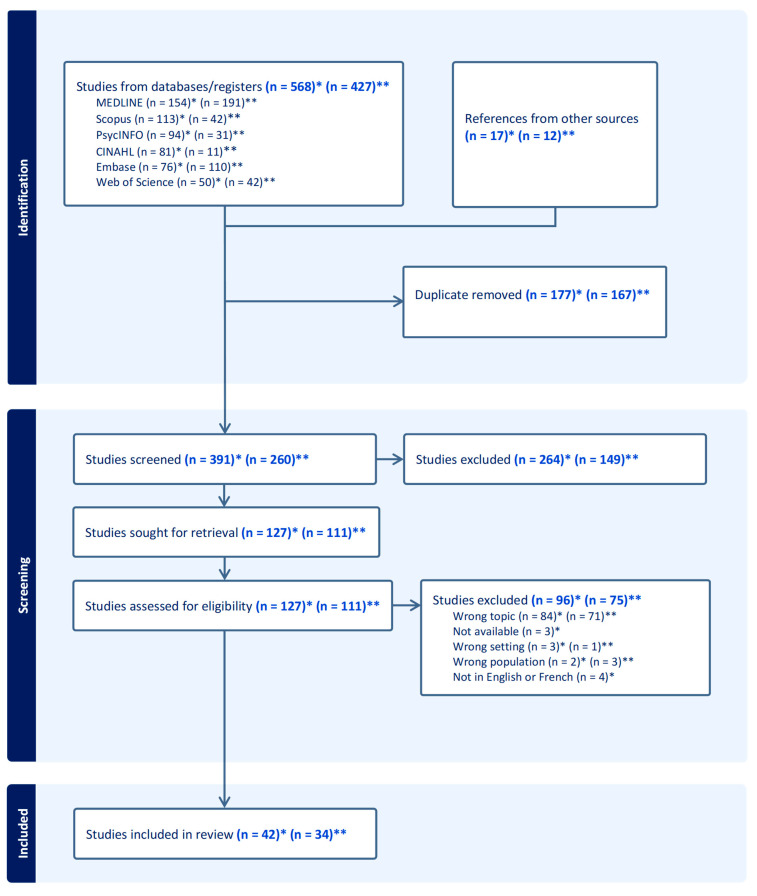
PRISMA flowchart. Note. * First literature search. ** Second literature search.

Part 1. For the first literature search, studies were retrieved from databases (n = 568) and through hand-searching relevant materials and bibliographic citations from other sources (n = 17). These articles were then uploaded to Covidence for duplicate removal. Titles and abstracts were screened in Covidence, and the remaining articles were transferred to Zotero for full-text review (n = 127). Based on the inclusion and exclusion criteria, 96 studies were excluded, resulting in 42 studies included in the review (see Figure 1). The characteristics of the included studies can be found in Table 1. Most studies originated from high-income countries, with 40% (17/42) conducted in the United States (U.S.). All selected articles were published in English, and publication years ranged from 2001 to 2024. Following data extraction and organization into a data analysis table (see Table S1 in Supplementary Materials), four main themes were identified through inductive analysis: (1) food insecurity and mental health; (2) obesity and mental health; (3) diet quality and mental health; and (4) gut microbiome and mental health.

Part 2. For the second literature search, studies from databases (n = 427) and hand searches (n = 12) were processed similarly to the first search, with duplicate articles removed and titles and abstracts screened in Covidence. Full-text screening in Zotero (n = 111) led to the exclusion of 75 studies and the final inclusion of 34 articles (see Figure 1). Table 2 summarizes the characteristics of the included studies. All selected studies were published in English, with the majority (65%; 22/34) conducted in Canada. Although Canadian studies were prioritized during the screening process, relevant studies from other countries were also included due to the limited data on Canadian immigrants. Publication years ranged from 2003 to 2024, with most of them (82%) published within the last decade (2014–2024).

#### 3.1.1. Part 1: Bidirectional Concept Between Nutrition and Mental Health

##### Food Insecurity and Mental Health

Food insecurity and mental health are intricately linked, with evidence suggesting a bidirectional relationship mediated by factors such as comorbidities, poverty, and societal stigma. A longitudinal U.S. household survey (N = 7946) identified significant bidirectional associations between food insecurity and psychological distress [25]. Additional longitudinal research found mutual relationships between food insecurity and maternal depression [26] and between household food insecurity and depression [24]. Furthermore, improvements in mental health were associated with better food security, whereas food insecurity depleted psychosocial coping resources and increased psychological distress [21,29]. A systematic review highlighted that food insecurity increased the risk of poor emotional health, which in turn raised the likelihood of food insecurity [20]. A meta-analysis reported that people with mental illnesses were 2.71 times more likely to experience food insecurity than those without psychological illnesses [28]. Cohort studies found that prenatal mental health issues increased food insecurity risks, and vice versa [18]. Among low-income mothers, depression emerged as a significant predictor of food insecurity [23]. A U.S. cross-sectional study (N = 25,444) found strong associations between food insecurity, severe mental illness, stigma, and emotional distress [19], and food insecurity was significantly more prevalent in people with mood disorders compared to the general population in a Canadian study [22]. Meta-analyses of cross-sectional data suggested individuals with severe food insecurity were over twice as likely to experience depression as those who were food secure [27].

##### Obesity and Mental Health 

Evidence suggests a complex, bidirectional relationship between obesity and mental health. A systematic review with meta-analysis found that individuals with obesity had a 55% increased risk of developing depression, while individuals with depression were 58% more likely to suffer from obesity [36]. Among 65,955 middle-aged and older women followed prospectively, obesity and depression demonstrated a bidirectional relationship [38]. A longitudinal cohort with a 20-year follow-up (N = 1656) found that obesity predicted depressive symptoms in women, while depression predicted later weight gain in men [35]. In a prospective study of 7108 individuals, depression and obesity reciprocally influenced each other over time [42]. A three-decade prospective study (N = 544) found that obesity significantly increased the odds for generalized anxiety disorder and major depressive disorder (MDD) by 6.27 and 5.25 times, respectively, after adjusting for other risk factors [34]. Cross-sectional studies revealed consistent associations between obesity and depression across demographics in middle-aged women (N = 4641) [41] and identified obesity as an independent risk factor for depression (N = 10,348) [37]. Among adolescents in a prospective cohort study (N = 9374), baseline depressed mood predicted obesity at follow-up [32], while a longitudinal study (N = 1037) found late-adolescent depression in girls associated with later obesity [40]. In a longitudinal study of 1554 African American and White females in the U.S., Franko et al. (2005) reported that depressive symptoms during adolescence were linked to an increased risk of obesity and higher BMI in adulthood, after controlling for prior BMI and parental education levels [31]. Although African American females had higher BMI and obesity rates overall, the depression-obesity link remained consistent across racial groups [31]. Childhood depression was also linked to adult obesity. A case-control study of children aged 6–17 years with major depression (N = 177) found higher adult body mass index (BMI) compared to controls [39]. Similarly, a 21-year cohort (N = 591) identified childhood depression as a predictor of adult obesity, particularly in women [33].

##### Diet Quality and Mental Health 

Research highlights a bidirectional relationship between mental health and diet quality, with each influencing the other. Analyses from a five-year prospective cohort (N = 3486) found that a processed food dietary pattern increased depression risk, while a whole food pattern offered protective benefits [43]. Prospective studies suggested that the Mediterranean diet was protectively linked to reduced depression risk, while fast food or commercial baked goods consumption was associated with an increased risk of depression [52,53]. Findings from another prospective study (N = 3040) indicated that improvements in diet quality led to better mental health during the follow-up period, whereas a decline in diet quality was linked to worsening psychological well-being [47]. A six-year retrospective cohort study (N = 22,385) identified mutual associations between dietary diversification and depressive symptoms [45]. Findings from a systematic review and meta-analysis suggested that high intakes of vegetables, fruits, fish, and whole grains were significantly associated with reduced odds of depression [50]. Cross-sectional findings suggested a bidirectional link between diet quality and mental health conditions [44]. “Western” diets, characterized by processed food consumption, were correlated with increased risks of depression and anxiety, whereas whole-food diets were associated with decreased risks [46,48,49,51].

##### Gut Microbiome and Mental Health 

Research into the effects of dietary changes on the gut–brain axis has improved our knowledge of the dietary role in this two-way communication. Findings from a pre-clinical trial provided insights about the bidirectional communication between the brain and the gut and how the neuroendocrine system acts as a key component that can be affected by changes in the enteric microbiota via food intake, including probiotics [55]. Mice transplanted with fecal microbiota from individuals with MDD exhibited depression-like symptoms compared to controls in a pre-clinical investigation, indicating that dysbiosis of the gut microbiome could play a causal role in the onset of depression [59]. Other findings from a pre-clinical study suggest that gut microbiota can influence several aspects of brain function, and its disruption may contribute to the development of neuropsychiatric disorders [56]. Clinical trials suggested that probiotics and dietary interventions may serve as complementary treatments for mental health conditions, influencing anxiety, stress, and depression through their effects on gut microbiota and the enteric and central nervous system [54,57,58].

#### 3.1.2. Part 2: Nutritional and Mental Health of Immigrants in Canada and the HIE

##### Food Insecurity and Immigrants 

The connections between food insecurity and immigrants’ psychological well-being appear to be multifaceted and to influence each other. A multi-methodological study in Ontario found that the impacts of immigrants’ food insecurity and dietary habits on their mental health were complex and interrelated, involving factors such as age, gender, religion, socioeconomic status, parenting, disabilities, and location of residence [69]. A cross-sectional study of 190 Ottawa-based immigrant mothers from sub-Saharan Africa and the Caribbean reported a high rate of food insecurity (45%) [85]. Additionally, length of residence in Canada strongly affected household food insecurity, with recent migrants (≤5 years) being more affected compared to those who had lived in Canada for more than a decade [85]. Qualitative findings also underscored the cultural dimensions of food insecurity in Canadian immigrants, as many newcomers faced barriers to obtaining culturally appropriate foods [79]. Consuming traditional ethnic foods was linked to improved mental health outcomes in immigrants, emphasizing the importance of dietary familiarity for psychological well-being [3].

##### Obesity and Immigrants 

Empirical evidence indicates that immigrants to Canada generally have lower rates of overweight and obesity upon arrival, reflecting the HIE [77]. Nevertheless, other factors associated with the process of acculturation and adherence to ethnic social norms can contribute to gradual weight gain over time [60,67,77,84]. According to a U.S. cross-sectional study (N = 32,374), after controlling for age, sociodemographic, and lifestyle factors, immigrants who had been living in the U.S. for over 10 years had an increase in BMI [71], while immigrants who had been living for 15 years or more had an obesity rate comparable to that of U.S.-born adults [71]. Evidence from a cross-sectional study suggested that social networks within ethnic communities can provide a buffer against the lifestyle changes associated with Canadian living, helping to reduce the risk of excessive weight gain over time [77]. Conversely, qualitative research highlighted cultural practices of people from North Africa and the Middle East, where guests are encouraged to eat abundantly as a sign of respect, a practice that can promote overeating and lead to obesity [67]. Findings from qualitative research with sub-Saharan African immigrants revealed that gaining weight post-immigration is commonly regarded as a positive indicator of prosperity and is often encouraged by family members in their countries of origin [60]. In this context, slimness in women is sometimes viewed negatively, while larger body sizes are associated with health and attractiveness [60]. Similarly, for men, a larger belly is often perceived as a symbol of wealth [60].

##### Diet Quality and Immigrants 

Many newcomers to Canada wish to preserve their healthy eating practices upon arrival, yet they frequently face several barriers. Qualitative studies indicated that immigrant families often struggle with maintaining their traditional, nutritious diets due to insufficient guidance on navigating the Canadian food environment, demanding schedules, and pressure from children to consume fast food, which can contribute to poorer dietary choices [75,81]. According to a cross-sectional study in Canada (N = 22,480), there was a significant decline in diet quality for immigrants 2 to 5 years post-settlement. Moreover, all immigrant groups reported poorer mental health than native-born Canadians, with recent immigrants facing higher mental health challenges and greater food insecurity within their first five years post-settlement. While food insecurity may play a role in both issues, the cross-sectional nature of the study prevents establishing causal relationships [66]. Results from another Canadian cross-sectional study (N = 15,595) observed that living in neighborhoods with a high concentration of immigrants and resources tailored to diverse groups may help individuals preserve healthy eating practices from their home countries [70].

##### Gut Microbiome and Immigrants 

The gut microbiota of immigrants to Western countries appears to undergo a transformation as a consequence of dietary acculturation, which is linked to health issues over time. A longitudinal cohort study (N = 550) indicated that immigrating to the U.S. caused important changes to the gut microbiome, such as loss of microbial diversity, loss of native strains, and loss of fiber breakdown capabilities [87]. These alterations occurred immediately upon arrival and were exacerbated in individuals with obesity and second-generation immigrants born in the U.S. [87]. According to a prospective cohort study (N = 2640), the gut microbiome diversity of newcomers was shown to be altered following immigration and was linked to an increased risk of obesity, possibly as a result of a westernized diet acculturation [88]. Results from a Canadian cohort study (N = 2,144,660) showed that immigrants to Canada had a lower incidence of inflammatory bowel disease (IBD); however, within one generation, the children of immigrants assumed the same incidence risk as nonimmigrants [63]. A prospective cohort study (N = 16,415) in the U.S. found that greater dietary acculturation to the Western diet was associated with an increased risk of CVD among Hispanic and Latino adults, potentially related to changes in gastrointestinal microbiota [89]. According to cross-sectional data, alteration in the gut microbiota was associated with symptoms of depression among Chinese and Korean immigrants in the U.S. [73].

##### Mental Health and Immigrants

Immigrants from non-Western backgrounds often experience a decline in mental health following migration. In a ten-year large retrospective cohort study in Ontario, Canada (N = 4,284,694), all refugees, especially people from East Africa and South Asia, as well as immigrants from the Caribbean and Bermuda, had a greater incidence of psychotic disorders compared to the general population, whereas immigrants from Europe and East Asia had lower risks [62]. Refugee status independently predicted a heightened risk of psychotic disorders across all migrants [62]. Furthermore, a Canadian retrospective cohort study (N = 106,080) indicated a significant decrease in mental health among migrants; however, there was no substantial change in the overall utilization of mental health services within and across these cohorts [76]. Findings from a three-year prospective cohort study in the Netherlands (N = 4076) highlighted that perceived discrimination might contribute to the elevated rates of psychotic disorders in immigrants from visible minority populations [74]. A systematic review provided robust evidence of a decline in mental health among newcomers in the years after immigration [68]. Results from a systematic meta-analysis showed that the increased risk of schizophrenia and similar conditions persisted across generations of immigrants, suggesting the importance of post-migration factors and the role of the social context in mediating this risk [64]. A qualitative study of 231 West African immigrant women found that those who had undergone female genital mutilation (FGM) experienced significantly lower well-being, higher psychological distress, and post-traumatic stress disorder (PTSD) after immigration [78]. Additionally, a systematic review found that migrant women with FGM were more likely to exhibit reduced health-seeking behaviors and increased negative experiences with healthcare services [83].

##### Healthy Immigrant Effect

Overall, there is evidence that immigrants in Canada benefit from the HIE [2]. Research data, however, may not reflect the whole picture due to differences in immigrant admission classes; those admitted in the economic class usually display better health at baseline [7,80,86]. Additionally, underdiagnosis of mental health illnesses among racialized migrants and the generally better health of White newcomers may impact the accuracy of health data interpretation [4,7].

## 4. Discussion

### 4.1. Discussion of Main Findings

The purpose of this integrative review was to synthesize existing literature on the bidirectional relationship between nutrition and mental health and to explore its implications for the mental and physical health of immigrants in Canada. The review also examined the HIE in Canada, including a focus on immigrants’ mental health. Four overarching themes emerged from the literature: (1) food insecurity and mental health, (2) obesity and mental health, (3) diet quality and mental health, and (4) the gut microbiome and mental health. These themes provide valuable insights into the complex biological and sociocultural interactions that shape the health and well-being of Canadian immigrants through the lens of nutrition and mental health.

Evidence supports the presence of the HIE in Canada, particularly among economic-class migrants who arrive with better overall health than the Canadian-born population. However, research indicates that this advantage diminishes over time, with physical and mental health declining, especially among racialized groups [2,4,7,80,86,90,91]. Various social determinants of health, including employment status, income level, access to social support networks, culturally appropriate healthcare, language proficiency, acculturation challenges, and experiences of discrimination, can all contribute to this decline [80,91,92,93,94,95,96,97,98]. However, much of the existing evidence supporting the HIE is based on self-reported health data, which may introduce bias, as responses can be shaped by sociocultural norms, stigma surrounding mental health, recall limitations, and language barriers [82,90]. Furthermore, many studies categorize immigrants as a homogeneous group, overlooking important differences in religion, country of origin, and language [95]. Immigrant experiences also vary widely across different provinces, regions, and urban centers in Canada, affecting their integration and access to resources [99]. Health disparities are also observed across immigrant admission categories [82,90]. Canada’s immigration system is highly regulated and operates under three primary admission categories: economic, family sponsorship, and refugee programs [100]. More than half of immigrants fall under the “Economic Immigrants” class, which includes skilled workers and investors. “Family Sponsorship” accounts for approximately a quarter of newcomers, allowing citizens and permanent residents to sponsor close relatives. “Refugees”, granted residency due to persecution risks, comprise around 10%, although this figure fluctuates [100,101,102]. Studies suggest that economic immigrants tend to have better health outcomes, followed by family-class immigrants, while refugees often experience the greatest declines in health [62,82,90]. Nonetheless, all immigrant classes face earnings disadvantages compared to Canadian-born workers [103]. Individuals without legal immigration status in Canada are among the most vulnerable populations, facing substantial barriers to accessing essential services [104,105]. Their exclusion from healthcare, social programs, and legal protections directly affects their health and well-being [104,105]. Consequently, immigration status is a crucial determinant of both mental health and dietary behaviors, shaped by broader social and economic factors [104,105]. Refugees often carry the burden of trauma and unmet health needs from their time in refugee camps and long migration journeys, frequently leading to chronic health conditions, both physical and mental [62,98,106]. Only about 30% of refugees speak one of Canada’s official languages before arrival, which complicates their integration and exacerbates their susceptibility to health challenges [98,106]. A Canadian study found that refugee children had a higher prevalence of stunted growth than immigrant children [107]. Other research findings pointed to several nutritional deficiencies commonly observed in refugee children, including vitamin D, vitamin B12, folate, and iron [108,109,110]. Ethnic minority girls, particularly refugees, faced greater risks of poor bone development due to low vitamin D levels, with children from the Middle East, Asia, and Africa at higher risk of deficiency [111]. Since early bone growth affects osteoporosis risk, these deficiencies may lead to long-term health issues [111]. Additionally, widespread iron deficiency and anemia have been observed among newly arrived refugees in Canada, with women exhibiting rates nearly three times higher than those of Canadian-born females [112].

Findings from this review suggest that food insecurity and mental well-being may impact each other and may be linked to cumulative factors such as comorbidities, societal stigma, and poverty [18,19,20,21,22,23,24,25,26,27,28,29]. Moreover, economic instability, unstable housing and employment, as well as cultural dimensions, such as accessing and preparing food that aligns with traditional practices, further complicate food security for many newcomers [3,79,113]. Language barriers, limited social networks, and fear of racial discrimination can further restrict their ability to seek financial or food aid [3,114]. These cultural and psychosocial stressors may amplify the psychological impact of food insecurity and hunger, leading to complex mental health issues [3]. Canadian studies indicate that food insecurity during childhood can lead to increased risks for physical and mental health problems, including depression and asthma, later in adolescence and early adulthood [115,116]. Immigrants to Canada, especially those recently arrived, refugees, and single parents are particularly vulnerable to food insecurity [3,66,69,79,85,117]. The adjustment to the North American food system often brings challenges that negatively affect immigrants, including a higher risk of loss of cultural identity, depression, household food insecurity, and poor nutritional outcomes [79]. This cycle is often both bidirectional and perpetuating [79]. Research data also indicate that visible minority groups in Canada (excluding Indigenous and White populations) are more likely to experience economic instability, leaving them more vulnerable to financial challenges and food insecurity compared to non-racialized Canadians [118].

According to the findings of this review, relationships between depression and obesity can be bidirectional and complex [30,31,32,33,34,35,36,37,38,39,40,41,42]. Immigrants to Canada are generally less likely to be overweight or obese upon arrival compared to Canadian-born individuals, consistent with the HIE [77]. However, other factors related to acculturation and adherence to traditional ethnic social norms can lead to weight gain over time [60,67,77,84]. This is consistent with trends observed in the U.S., where studies found a correlation between rising obesity rates among immigrants and longer duration of residence, after controlling for sociodemographic variables [71,119,120]. Evidence suggests that dietary habits can influence mood, while stress-related mental health conditions can lead to changes in eating behaviors, contributing to weight gain [121]. Meanwhile, obesity can elevate the risk of depression, potentially through its association with chronic inflammation and other physiological mechanisms [122,123,124]. Chronic inflammation has been shown to aggravate depression and other psychiatric conditions, while depression itself can induce inflammatory responses [122]. Women appear particularly vulnerable to the effects of obesity-related depression compared to men [35,42]. Additionally, a number of studies have shown that emotional eating, which is the practice of increasing food intake in response to stressful or negative emotions, is associated with a higher risk of depression and obesity [42,125,126,127]. Studies, however, indicate that the relationship between obesity and depression varies across racial and ethnic groups [128,129] and is influenced by social and demographic variables [130,131]. Cultural factors, such as body image norms, suggest that Black women, for example, may experience less societal pressure and adhere to different body ideals compared to White women [114,131,132]. In contrast, other studies report a consistent association between obesity and depression among women, regardless of race [31,133].

Results from this review suggest that the quality of an individual’s diet can influence mental well-being in a bidirectional manner [43,44,45,46,47,48,49,50,51,52,53]. While many immigrants strive to maintain their healthy dietary habits after arriving in Canada, they often encounter challenges that can negatively impact the quality of their diet [66,70,75,81]. Socioeconomic challenges, such as financial pressures and language barriers, amplify the mental health and nutritional risks for migrant populations [3,134]. Newcomers also frequently report difficulties adjusting to their new, unfamiliar food system, including large supermarket shopping, leaving many of them unprepared to make informed dietary choices [66,75,135,136]. Qualitative evidence indicates that strategies promoting self-efficacy and the preservation of healthy food traditions enhanced immigrants’ ability to adapt to Canada’s food environment while maintaining traditional eating habits, fostering successful resettlement [72]. Consistent with previous findings, ethnic cuisines, often rich in nutrients, have been shown to enhance both physical and mental health by enabling immigrants to maintain balanced diets in unfamiliar environments [3,113,114,137]. Empirical evidence suggests that higher fruit and vegetable consumption is associated with decreased levels of depression and psychological distress [3,138]. Research into nutritional approaches to depression management has explored specific nutrient supplementation such as omega-3 fatty acids, B vitamins, magnesium, zinc, and amino acids as potential adjuvant therapies, although findings have been inconsistent or limited [121,139,140,141,142]. Research, including systematic reviews and meta-analyses, indicates that diets such as the Mediterranean diet, rich in vegetables, fruits, whole grains, and fish, may offer protective effects against depression [43,46,47,48,50,51,52,53]. By contrast, Western diets characterized by processed foods, refined sugars, and unhealthy fats have been linked to an increased risk of depression [50,141,143,144,145]. However, most of the meta-analyses relied heavily on cross-sectional data, making it challenging to establish the temporal sequence of the diet-depression relationship. Additionally, high heterogeneity in these studies, arising from variations in dietary assessments and adjustments for confounding variables, has further complicated the interpretation of data.

Findings from this review underscore the profound influence of dietary factors on the gut microbiome, brain structure and function, and mental well-being. Although much of the research in this area has been conducted using animal models and some inconsistencies exist in the findings, there is a discernible trend suggesting that dietary interventions directly affect gut flora. In turn, this mediates the bidirectional communication between the gut and the brain, positioning the gut microbiome as a key factor in the relationship between diet and depression [146,147,148]. Dietary intake of prebiotics and probiotics from dietary fiber, fermented foods, and polyphenols has been shown to stimulate beneficial intestinal microbial growth and gut–brain communication, which could be involved in improving mental illnesses [122,146,148,149,150]. For immigrants, adapting to Western diets often disrupts gut microbiota, increasing the likelihood of obesity and other health risks over time [73,87,88,151,152].

Finally, according to the World Health Organization (WHO), depression and other mental illnesses can have a profound impact on health, contributing to daily life challenges, an elevated risk of suicide, and strong associations with physical conditions such as cardiovascular disease, cancer, type 2 diabetes, and respiratory disorders [153]. Research highlights that unemployment and lower household income are crucial factors affecting mental health, with economic hardship recognized as a major determinant of health, contributing to disparities such as food insecurity, poor diet quality, obesity, and diet-related chronic diseases like type 2 diabetes [154,155,156,157,158,159,160]. Several studies have indicated that migrants from non-Western backgrounds often experience a decline in mental health after immigration and are at increased risk for mental health issues, including psychotic disorders and PTSD [61,62,64,66,68,74,76,78,82,83,161]. Racialized immigrants and refugees are particularly vulnerable to these challenges due to factors such as socioeconomic hardship, systemic racism, social exclusion, and job market disadvantages [62,82,91,161]. Although employment barriers tend to improve in subsequent generations, disparities persist for some groups [162]. A less recognized but potentially critical factor impacting the mental health of immigrant women is a history of FGM. According to the WHO, FGM remains common in 30 countries across Asia, Africa, and the Middle East, with over 230 million girls and women globally having undergone the practice, typically between infancy and age 15 [163]. Emerging evidence links FGM to higher rates of depression, anxiety, and PTSD among affected immigrant women [78,83]. Additionally, these women are often hesitant to seek medical attention, partly due to negative experiences with healthcare providers who may display visible discomfort or a lack of understanding, leading to feelings of shame and disrespect, according to qualitative findings [83]. Given the large influx of immigrants to Canada from regions where FGM remains prevalent, it is crucial to acknowledge and address the potential impacts of this often-overlooked aspect of immigrant women’s health.

### 4.2. Recommendations for Policy and Research 

Addressing social determinants of health through upstream public health measures is essential for preventing physical and mental health deterioration among Canadian immigrants. To ensure equitable health care, it is essential to address structural barriers and enhance access to targeted mental health and social support services. These resources are particularly necessary for high-risk groups, including refugees and family-class immigrants, to support their successful integration into Canadian society. Additionally, to prevent a decline in mental health, these services should continue to be accessible for several years following settlement.

The strong interplay between mental health and nutrition highlights the need to integrate dietary considerations into standard depression treatment. Further clinical research into the gut microbiota may also offer novel insights into stress-related psychiatric disorder treatments. Additionally, given the link between depression and obesity, preventive strategies, such as systematic depression screening for high-risk individuals, are crucial for reducing long-term health risks. Moreover, to improve health outcomes, healthcare professionals should receive training in culturally competent and empathetic care, ensuring that services are accessible and appropriate for diverse immigrant communities.

Additionally, policies that strengthen social assistance, employment opportunities, affordable housing, and childcare could help enhance overall well-being by improving mental health, food security, and diet quality. Vulnerable populations, including racialized groups, refugees, and single-parent households, require close monitoring for food insecurity, particularly during the early years of resettlement.

Community-based initiatives, including culturally inclusive food programs, nutrition education, and food-related social programs, have shown promise in promoting social cohesion and reducing food insecurity. Programs such as community kitchens and culturally adapted cooking classes provide not only nutritional support but can also foster a sense of belonging among immigrant populations.

Future research should focus on identifying disparities among immigrant subgroups, particularly those differentiated by admission class, ethnicity, and socioeconomic status. Investigating the intersections of nutrition, culture, and mental health will provide valuable insights into the broader factors shaping immigrant health outcomes in Canada and worldwide. Examining mental health determinants and protective factors, such as social cohesion and culturally relevant resources, would help inform the development of targeted nutrition interventions. Additionally, exploring how immigrants engage with informal and non-mainstream mental health care, such as social networks and faith-based services, would be useful in shaping more inclusive and effective support strategies.

### 4.3. Strengths and Limitations 

The present review has both strengths and limitations. Among its strengths is the inclusion of a wide range of peer-reviewed published studies, encompassing various methodological designs, allowing for a comprehensive analysis of the topic. Moreover, two literature searches were conducted, providing a deeper and richer examination of the themes identified in the initial search. Additionally, all included studies were assessed to be of medium to high quality using the MMAT tool. On the other hand, the process of combining the results of several study designs can be complex and can potentially induce bias. The risk of bias in this review was mitigated by the methodological frameworks and the systematic, rigorous approach followed by both researchers. Another limitation is that studies often group all immigrant populations together without differentiating between admission classes, which can distort data interpretation by overlooking the distinct characteristics of these groups. Furthermore, while the second literature search prioritized Canadian studies, the inclusion of research from other locations due to limited available data may affect generalizability. Additionally, while the first literature search on the nutrition-mental health relationship was not focused on any specific location, most included studies originated from high-income countries, potentially limiting applicability to developing countries. Finally, only literature published in English was included, and other relevant studies in different languages may be missing.

## 5. Conclusions

The findings of this integrative review suggest that Canadian immigrants’ nutritional and mental health are influenced by complex bidirectional interactions. These processes can occur through direct or indirect pathways of varying complexity. While most immigrants arrive in Canada in good health, many experience a decline in their physical and mental well-being over time. Several recent newcomers face food insecurity and struggle to eat a healthy diet. Besides, sociocultural and economic factors may contribute to weight gain, while dietary changes following immigration may negatively impact the gut microbiome, potentially leading to adverse health outcomes. To prevent the decline in immigrant health in Canada and worldwide, it is essential to address this issue from multiple perspectives, as the relationship between diet and mental health is often shaped by mutually reinforcing dynamics. The intersectionality of these determinants plays a crucial role in shaping the health outcomes of immigrants. Considering that newcomers will continue to make up a large share of the Canadian population in the upcoming years, the health of immigrants and their offspring is of primary importance, as it will help shape the overall health landscape of the country. Therefore, further research on the interplay between nutrition and mental health among immigrants is needed, alongside the development of public health strategies that will protect, promote, and enhance their health so they can live with dignity and reach their full potential. 

## Figures and Tables

**Table 1:** Characteristics of the studies included from the first literature search.

Theme	#	Author/s, Year	Country	Design	Study Population	Main Findings of Relevance	MMAT Score
Food Insecurity and Mental Health	1	Abrahams and Lund (2022) [18]	South-Africa	Cohort study	Perinatal women (N = 635)	FI was significantly associated with CMD and psychological distress, while CMD was significantly associated with experiencing psychological distress and FI.	5/5
	2	Berrett-Abebe and Reed (2024) [19]	U.S.	Cross-sectional study. SDA.	2016 Medical Expenditure Panel Survey (N = 25,444)	There are likely bidirectional relationships between FI and chronic conditions including serious mental illness.	5/5
	3	Bruening et al. (2017) [20]	U.S.	Systematic narrative review of longitudinal research	Children and adults (12 included studies)	Findings suggest a bidirectional association whereby FI increases the risk of poor emotional health, and poor emotional health increases the risk of FI.	5/5
	4	Ciciurkaite and Brown (2022) [21]	U.S.	Longitudinal study	Florida residents (N = 1488)	FI is associated with diminished psychosocial coping resources, which leads to greater psychological distress.	5/5
	5	Davison and Kaplan (2015) [22]	Canada	Cross-sectional study. SDA.	Adults (N = 97)	FI was significantly more prevalent in the adults with mood disorders in comparison to the general population. There was significant association between FI and mania symptoms.	4/5
	6	Garg et al. (2015) [23]	U.S.	Cohort study	Low-income mothers (N = 2917)	Results suggest that maternal depression is an independent risk factor for household FI in low-income families with young children.	5/5
	7	Huddleston-Casas et al. (2009) [24]	U.S.	Longitudinal study	Women (N = 413)	Results indicated that the causal relationship between household FI and depression is bidirectional.	4/5
	8	Kim-Mozeleski et al. (2021) [25]	U.S.	Prospective study	Adults (N = 7946)	Results showed significant bidirectional associations between FI and psychological distress.	4/5
	9	Reesor-Oyer et al. (2021) [26]	U.S.	Longitudinal study	Mothers of young children (N = 4897)	Results show a bidirectional relationship between FI and maternal depression.	4/5
	10	Smith et al. (2021) [27]	UK	Meta-analysis of cross-sectional data	Adults (N = 34,129)	Those who suffer from severe FI compared with no FI were over two times more likely to have depression.	4/5
	11	Teasdale et al. (2023) [28]	Australia	Systematic review and meta-analysis	Adults (31 included studies)	People with severe mental illness were 2.71 times more likely to report FI than the comparator group.	4/5
	12	Tirfessa et al. (2020) [29]	Ethiopia	Longitudinal study	Adults (N = 512)	Significant improvement in FI in households of people with severe mental illness where an integrated mental healthcare plan was implemented.	5/5
Obesity and Mental Health	1	de Wit et al. (2010) [30]	The Netherlands	Meta-analysis of cross-sectional studies	General population (17 included studies)	Results showed a significant positive association between depression and obesity in the general population, which appeared to be more marked among women.	5/5
	2	Franko et al. (2005) [31]	U.S.	Longitudinal study	Adolescent girls (N = 1554)	Depressive symptoms in adolescence appear to be predictive of obesity and elevated BMI in early adulthood.	5/5
	3	Goodman and Whitaker (2002) [32]	U.S.	Prospective cohort study	Adolescents (N = 9374)	Having depressed mood at baseline independently predicted obesity at follow-up.	4/5
	4	Hasler et al. (2005) [33]	Switzerland	Cohort study	Young adults (N = 591)	Results show a strong longitudinal association between childhood depressive symptoms on adult BMI increasing with age leading to a considerable increase in the incidence of female obesity.	5/5
	5	Kasen et al. (2008) [34]	U.S.	Prospective study	Mothers (N = 544)	Obesity may have long-term implications for mental distress in women at a clinical level over the adult years.	5/5
	6	Konttinen et al. (2014) [35]	Finland	Longitudinal study	Adults (N = 1656)	Evidence showed that women with excess body weight were more likely to have increased symptoms of depression 10 years later. Symptoms of depression are important predictors of later weight gain also among men.	4/5
	7	Luppino et al. (2010) [36]	The Netherlands	Systematic review and meta-analysis of longitudinal studies	Children and adults (15 included studies)	Results show bidirectional associations between depression and obesity.	5/5
	8	Ma and Xiao (2010) [37]	U.S.	Cross-sectional study. SDA.	Women (N = 10,348)	Finding suggests that degree of obesity is an independent risk factor for depression	4/5
	9	Pan et al. (2012) [38]	U.S.	Prospective cohort study	Women (N = 65,955)	This cohort study suggest a bidirectional association between depression and obesity in middle-aged and elderly women.	5/5
	10	Pine et al. (2001) [39]	U.S.	Prospective case-control study	Children (N = 177)	Depressed children exhibit a larger BMI as adults than do nondepressed comparisons.	5/5
	11	Richardson et al. (2003) [40]	U.S.	Longitudinal cohort study	Children, Adults (N = 1037)	Depressed late adolescent girls were at a greater than 2-fold increased risk for obesity in adulthood compared with their nondepressed female peers	4/5
	12	Simon et al. (2008) [41]	U.S.	Cross-sectional study. Q.	Women (N = 4641)	Among middle-aged women, obesity is strongly associated with depression.	5/5
	13	Vittengl (2018) [42]	U.S.	Prospective study	Adults (N = 7108)	Among women but not men, depression predicted increased obesity, and obesity predicted increased depression in bidirectional ways, over a period of 18 years.	4/5
Diet Quality and Mental Health	1	Akbaraly et al. (2009) [43]	UK	Prospective cohort study	Whitehall II cohort (N = 3486)	A whole food pattern had lower odds of depression while high consumption of processed food was associated with an increased odd of depression.	5/5
	2	Brierley et al. (2021) [44]	Australia	Cross-sectional study. Q.	Member of general population (Australian n = 880, US n = 829)	Psychological distress was somewhat important in explaining the bidirectional relationship between diet quality and mental health disorders.	5/5
	3	Dong et al. (2024) [45]	China	Retrospective cohort	Adult females (N = 22,385)	Results identified bidirectional associations between dietary diversity and depressive symptoms.	5/5
	4	Jacka et al. (2010) [46]	Australia	Cross-sectional study. Q.	Women (N = 1046)	A dietary pattern characterized by vegetables, fruit, meat, fish, and whole grains was associated with lower odds for major depression and for anxiety disorders. A “Western” diet was associated with higher depression.	5/5
	5	Jacka, F.N., et al. (2011) [47]	Australia	Prospective study	Adolescents (N = 3040)	Improvements in diet quality were mirrored by improvements in mental health, while reductions in diet quality were associated with declining psychological functioning	5/5
	6	Jacka et al. (2011) [48]	Australia	Cross-sectional study. Q.	Adults (N = 5731)	Those with better quality diets were less likely to be depressed, whereas a higher intake of processed and unhealthy foods was associated with increased anxiety.	5/5
	7	Kuczmarski et al. (2010) [49]	U.S.	Cross-sectional study. DR.	Adults (N = 1118)	Diet quality was significantly associated with reported symptoms of depression.	4/5
	8	Lai et al. (2014) [50]	Australia	Systematic review and meta-analysis	Adults (21 included studies)	Results suggest that high intakes of fruit, vegetables, fish, and whole grains may be associated with a reduced depression risk.	5/5
	9	Nanri et al. (2010) [51]	Japan	Cross-sectional study. S. and Q.	Adults (N = 521)	A healthy Japanese dietary pattern characterized by high intakes of vegetables, fruit, mushrooms and soy products was associated with fewer depressive symptoms.	5/5
	10	Sánchez-Villegas et al. (2009) [52]	Spain	Prospective cohort study	Adults (N = 10,094)	The Mediterranean dietary pattern is protectively associated with depression.	5/5
	11	Sánchez-Villegas et al. (2012) [53]	Spain	Prospective cohort study	Adults (N = 8964)	A higher risk of depression was associated with consumption of fast food.	5/5
Gut Microbiome and Mental Health	1	Benton et al. (2007) [54]	UK	Double-blind placebo-controlled trial with random allocation of subjects	Members of general population (N = 124)	The consumption of a probiotic-containing yoghurt improved the mood of those whose mood was initially poor.	4/5
	2	Bravo et al. (2011) [55]	Canada	Pre-clinical trial	Adult male BALB/c mice (N = 36)	Data indicate that in the bidirectional communication between the brain and the gut, the HPA axis is a key component that can be affected by changes in the enteric microbiota.	4/5
	3	Crumeyrolle-Arias et al. (2014) [56]	France	Pre-clinical trial	F344 male rats (N = 24)	Gut microbiota dysbiosis that can occur at various life stages may contribute to the development of neuropsychiatric disorders.	4/5
	4	Messaoudi et al. (2011) [57]	France	Pre-clinical and clinical trial	Male Wistar rats (N = 36); Adults (N = 55)	Daily administration of probiotics significantly reduced anxiety-like behavior in rats and alleviated psychological distress in volunteers.	5/5
	5	Rao et al. (2009) [58]	Canada	Randomized, double-blind, placebo-controlled pilot study	Patients with chronic fatigue syndrome (N = 39)	Results show a significant decrease in anxiety symptoms among those taking probiotics vs controls.	4/5
	6	Zheng et al. (2016) [59]	China	Pre-clinical trial	Stool samples from MDD adults (N = 121); GF mice	Fecal microbiota transplantation of GF mice with ‘depression microbiota’ derived from MDD patients resulted in depression-like behaviors compared with colonization with ‘healthy microbiota’ derived from healthy control individuals.	5/5

Note. CMD = common mental disorders; DR = dietary recalls; FI = food insecurity; GF = germ free; HPA = hypothalamic–pituitary–adrenal; MDD = major depressive disorder; MMAT = mixed methods appraisal tool; Q = questionnaire; S = survey; SDA = secondary data analysis; UK = United Kingdom; U.S. = United States.

**Table 2:** Characteristics of the studies included from the second literature search.

#	Author(s), Year	Country	Design	Study Population	Main Findings of Relevance	MMAT Score	Theme(s) *
1	Addo et al. (2021) [60]	Australia	Qualitative study. In-depth interviews.	Sub-Saharan African immigrants (N = 24)	Immigrants living in Western countries are often expected to gain weight post-immigration related to cultural expectation.	5/5	B
2	Amiri (2022) [61]	Iran	Systematic review and meta-analysis	Immigrants (78 included studies)	Results suggest that the prevalence of anxiety and PTSD in immigrants is high.	4/5	E
3	Anderson et al. (2015) [62]	Canada	Retrospective cohort study	Ontario residents (N = 4,284,694)	Results show high rates of psychotic disorders among refugees and certain groups of immigrants in Ontario.	5/5	E
4	Benchimol et al. (2015) [63]	Canada	Cohort study	Immigrants (N = 2,144,660)	Immigrants to Canada had lower incidence of IBD relative to nonimmigrants, but incidence increased with second generation.	5/5	D
5	Bourque et al. (2011) [64]	Canada	Systematic review and meta-analysis	Immigrants (21 included studies)	Increased risk of schizophrenia and related disorders among immigrants persists into the second generation.	5/5	E
6	Davison et al. (2017) [65]	Canada	Cross-sectional study. SDA.	Canadians (N = 15,546)	FI can contribute to over- and under-nutrition, nutrient excesses, disproportions, and deficiencies, eating disturbances, and mental illnesses.	4/5	A, C, E
7	Davison and Gondara (2021) [66]	Canada	Cross-sectional study. SDA.	Canadians (N = 22,480)	Compared to native-born Canadians, all immigrant groups were more likely to report poor MH and those ≤5 years post-settlement were more likely to report higher levels of FI. Mean levels of DQ were significantly lower in groups that were 2–5 years post-settlement.	5/5	A, C, E
8	Ebrahimi et al. (2024) [67]	Iran	Qualitative study. Interviews.	Women (N = 17)	Overeating is part of hospitality culture in many cultures.	5/5	B
9	Elshahat et al. (2022) [68]	Canada	Systematic review	Immigrants (58 included studies)	Evidence was found for a decline in immigrants’ MH over years.	5/5	E, F
10	Elshahat et al. (2023) [3]	Canada	Scoping review	Immigrants (63 included studies)	Unhealthful dietary acculturation to the Western lifestyle was associated with poor MH. FI and was significantly positively associated with depression and anxiety among immigrants.	4/5	A, C, E
11	Elshahat et al. (2024) [69]	Canada	Multi-methodological study.	Immigrants. Interviews (n = 50), photovoice (n = 26), Q (n = 60).	Participants reported various socioeconomic and structural barriers to nutritious eating, and experienced high FI, which was associated with negative MH.	4/5	A, C, E
12	Emerson and Carbert (2018) [70]	Canada	Cross-sectional study. SDA.	Immigrants (N = 15,595)	Higher immigrant density exhibited protective associations with odds of obesity for racial minority immigrant.	5/5	B, C
13	Fuller-Thomson et al. (2011) [4]	Canada	Longitudinal study	Immigrants (N = 7716)	The HIE suggests new immigrants to Canada enjoy better health, on average, than those born in Canada, yet the process of immigration is associated with health decline for some immigrants.	5/5	F
14	Goel et al. (2004) [71]	U.S.	Cross-sectional study. SDA.	US residents (N = 32,374)	Among different immigrant subgroups, number of years of residence in the US is associated with higher BMI.	5/5	B
15	Henderson and Slater (2019) [72]	Canada	Qualitative study	Immigrants, Q (n = 22), interviews (n = 16)	Evidence-based, culturally sensitive newcomer nutrition programs have the potential to mitigate the negative effects of FI and dietary acculturation for newcomers.	5/5	A, C
16	Hope et al. (2023) [73]	U.S.	Cross-sectional observational pilot study. S.	Immigrants (N = 20)	Associations between the gut microbial diversity and depressive symptoms were found among Chinese and Korean Americans.	4/5	D, E
17	Janssen et al. (2003) [74]	The Netherlands	Prospective cohort study	General population (N = 4076)	Perceived discrimination may induce delusional ideation and contribute to the high observed rates of psychotic disorder in exposed minority populations.	5/5	E
18	Lane and Vatanparast (2023) [75]	Canada	Qualitative study. In-depth interviews.	Immigrants (n = 22), professionals (n = 22)	Newcomers experience difficulties in keeping healthy dietary habits post-immigration.	5/5	C
19	Lin (2024) [5]	Canada	Cross-sectional study. SDA.	Canadians (N = 28,951)	The HIE in mental health may be mainly driven by the healthier profile of White immigrants and partly attributable to the under-detection of mental health conditions among racialized immigrants.	5/5	F
20	Lu and Ng (2019) [2]	Canada	Cross-sectional study. SDA.	Canadians (N = 130,000)	Results corroborate the existing literature on the presence of the HIE among immigrants.The HIE was found to be much weaker among refugees.	5/5	F
21	Mason et al. (2024) [76]	Canada	Retrospective cohort study	Canadians (N = 106,080)	Mental health declines the longer immigrants remain in Canada. Furthermore, some cohorts of immigrants have lower initial levels of mental health.	5/5	F
22	McDonald and Kennedy (2005) [77]	Canada	Cross-sectional study. SDA.	Canadians (N = 126,796)	For most immigrants to Canada, the probability of being overweight is lower on arrival than for comparable native-born Canadians, but increases gradually post-immigration.	4/5	B
23	Min et al. (2023) [78]	U.S.	Qualitative study. Interviews, S.	Immigrants (N = 231)	FGM significantly predicted lower well-being, higher psychological distress, and PTSD.	4/5	E
24	Moffat et al. (2017) [79]	Canada	Qualitative study. Interviews, FG.	Immigrants (n = 24), professionals (n = 22)	Cultural dimensions can affect FI, and FI may be more challenging for households that have lost parts of their traditional foodways.	5/5	A
25	Morassaei et al. (2022) [80]	Canada	Scoping review	Immigrants (27 included studies)	Health outcomes vary significantly across immigrant subgroups defined by the admission class through which they entered Canada.	4/5	F
26	Mycek et al. (2020) [81]	U.S.	Qualitative study. Interviews	Immigrants (n = 30, refugees (n = 8)	Immigrants arrive with healthy food practices, but they need resources to help them navigate the complicated food system.	4/5	C
27	Ng and Zhang (2020) [82]	Canada	Cross-sectional study. SDA.	Canadians (N = 208,919)	Immigrants’ mental health does not improve with time spent in Canada.	5/5	F
28	O’Neill and Pallitto (2021) [83]	Belgium	Systematic review of qualitative research.	Immigrants women (23 included studies)	Women with FGM have higher rates of depression, anxiety, and PTSD, as compared to women without FGM.	4/5	E
29	Su et al. (2020) [84]	Canada	Cross-sectional study. SDA.	Canadians (N = 109,875)	Obese respondents were more likely to suffer from depression than normal weight respondents among both immigrants and nonimmigrants.	5/5	B, E
30	Tarraf et al. (2018) [85]	Canada	Cross-sectional study. Interviews, Q.	Immigrant mothers (N = 190)	High rate of FI was found among participants. FI was associated with Caribbean origin, low education attainment, lone motherhood, living in Canada for ≤5 years.	5/5	A
31	Vang et al. (2017) [86]	Canada	Systematic review	Immigrants (78 included studies)	There is an absence of a uniform foreign-born health advantage in Canada, but HIE characterizes the majority of contemporary migrants in Canada.	5/5	F
32	Vangay et al. (2018) [87]	U.S.	Cross sectional and longitudinal cohorts	Immigrants (N = 569)	Migration from a non-Western country to the United States is associated with immediate loss of GM diversity and function.	4/5	D
33	Wang et al. (2021) [88]	U.S.	Prospective cohort study	Hispanic/Latino adults (N = 16,415)	U.S. immigration was associated with alterations in GM diversity.	5/5	D
34	Wang et al. (2024) [89]	U.S.	Prospective cohort study	Hispanic/Latino adults (N = 16,415)	Greater dietary acculturation was associated with elevated CVD risk, possibly through alterations in GM.	5/5	D

Note. CVD = cardiovascular disease; DQ = diet quality; FG = focus group; FGM = female genital mutilation; FI = food insecurity; GM = gut microbiome; IBD = inflammatory bowel disease; MH = mental health; MMAT = mixed methods appraisal tool; PTSD = post-traumatic stress disorder; Q = questionnaire; S = survey; SDA = secondary data analysis; U.S. = United States. *: A = food insecurity and immigrants; B = obesity and immigrants; C = diet quality and immigrants; D = gut microbiome and immigrants; E = mental health and immigrants; F = healthy immigrant effect.

## Data Availability

Not applicable.

## Supplementary Materials

The following supporting information can be downloaded at: https://www.mdpi.com/article/10.3390/healthcare13080850/s1, Table S1: Data Analysis Table.

## Abbreviations

BMI	Body mass index
CMD	Common mental disorders
CVD	Cardiovascular disease
DQ	Diet quality
DR	Dietary recalls
FG	Focus group
FGM	Female genital mutilation
FI	Food insecurity
GF	Germ free
GM	Gut microbiome
HIE	Healthy immigrant effect
HPA	Hypothalamic-pituitary-adrenal
IBD	Inflammatory bowel disease
MDD	Major depressive disorder
MH	Mental health
MMAT	Mixed Methods Appraisal Tool
N	Number
O	Obesity
PTSD	Post-traumatic stress disorder
Q	Questionnaire
S	Survey
SDA	Secondary data analysis
UK	United Kingdom
U.S.	United States
WHO	World Health Organization
