# Supplementary material for: Bidirectional Relationship Between Nutrition and Mental Health and Its Impact on the Health of Canadian Immigrants: An Integrative Review
Source: Healthcare (Basel). 2025 Apr 8;13(8):850. doi: 10.3390/healthcare13080850 (PMC12027039; doi:10.3390/healthcare13080850)
Supplement: Supplementary file 1 [file healthcare-13-00850-s001.zip › healthcare-3536864-supplementary.pdf]

**Table S1.** Data analysis of studies on the bidirectional relationship between nutrition and mental health (n=42).

| Data units | Summary of data units | Codes (essence of meaning of data units) | Subcategories |
|---|---|---|---|
| Food insecurity was significantly associated with race, several markers of multidimensional poverty (including employment status, receipt of grants and number of people per sleeping room), probable depression & anxiety, psychological distress and experiences of domestic violence; while probable depression & anxiety was significantly associated with experiencing psychological distress, food insecurity and domestic violence. (Abrahams & Lund, 2022) [18] | Food insecurity is associated with depression & anxiety, psychological distress, as well as poverty, race, domestic violence.<br>Depression & anxiety was significantly associated with experiencing psychological distress, food insecurity and domestic violence. | Bidirectional relationship between food insecurity and depression & anxiety. | Food insecurity and mental health. |
| Logistic regression models indicate strong, statistically significant relationships between FI and chronic conditions, including SMI. Theory and best evidence suggest complex and bidirectional pathways between FI and chronic conditions. Additionally, chronic conditions may strain household finances and resources due to an individual's inability to work and/or the high cost of medical care. This financial strain may force difficult decisions such as paying for medications versus paying for food. (Berrett-Abebe & Reed, 2024) [19] | Food insecurity may lead to developing chronic conditions including SMI. Chronic conditions including SMI may lead to food insecurity. | Bidirectional relationship between food insecurity and chronic illnesses, including serious mental illness. | Food insecurity and mental health. |
| The majority of studies included in the present review show significant associations between poor mental health and food insecurity and suggest a bidirectional association whereby food insecurity increases the risk of poor emotional health, and poor emotional health increases the risk of food insecurity. (Bruening et al., 2017) [20] | Findings suggest a bidirectional association whereby food insecurity increases the risk of poor emotional health, and poor emotional health increases the risk of food insecurity. | Bidirectional relationship between food insecurity and emotional health. | Food insecurity and mental health. |
| Findings reveal that greater food insecurity at wave 1 is associated with greater depressive symptoms at wave 2, lending credence to characterizations of food insecurity as a chronic stressor. This association is partly mediated by variation over the study period in social support and mastery. However, no significant mediating effects are observed for the social stress indicators assessed. (Ciciurkaite & Brown, 2022) [21] | Food insecurity is associated with diminished psychosocial coping resources, which leads to greater psychological distress. | Relationship between food insecurity and psychological distress. | Food insecurity and mental health. |

**Table S1.** Data analysis of studies on the bidirectional relationship between nutrition and mental health (n=42). *Continued*

| Data units | Summary of data units | Codes (essence of meaning of data units) | Subcategories |
|---|---|---|---|
| In comparison to the general population, food insecurity was significantly more prevalent in the adults with mood disorders. Respondents who were food-insecure had lower median intakes of carbohydrates and vitamin C ($p < 0.05$). In addition, a higher proportion of those reporting food insecurity had protein, folate, and zinc intakes below the DRI benchmark of potential inadequacy ($p < 0.05$). There was significant association between food insecurity and mania symptoms (adjusted prevalence ratio = 2.37, 95% CI 1.49–3.75, $p < 0.05$). (Davison & Kaplan, 2015) [22] | Food insecurity is associated with both nutritional and psychological health in adults with mood disorders. Investigation of interventions aimed at food security and income can help establish its role in enhancing mental health. | Relationship between food insecurity and mood disorders. | Food insecurity and mental health. |
| At baseline, 16% of mothers were depressed (raw score >9). Most mothers were white, unemployed, and born in the United States. The majority received Special Supplemental Nutrition Program for Women, Infants, and Children (WIC) (86%); 39% received Supplemental Nutrition Assistance Program (SNAP). At follow-up, 11.8% of mothers reported household food insecurity. In multivariable analysis, maternal depression at baseline was significantly associated with food insecurity at follow-up (adjusted odds ratio 1.50; 95% confidence interval 1.06–2.12). (Garg et al., 2015) [23] | Results suggest that maternal depression is an independent risk factor for household food insecurity in low income families with young children. Multidisciplinary interventions embedded within and outside the pediatric medical home should be developed to identify depressed mothers and link them to community-based mental health and food resources. | Relationship between food insecurity and maternal depression. | Food insecurity and mental health. |
| Findings based on the 184 subjects with complete data indicated that the causal relationship between household food insecurity and depression is bidirectional. Households headed by single mothers and households with incomes below the federal poverty line have higher rates of food insecurity than those in the general population. Minority households also have a higher rate of food insecurity. (Huddleston-Casas et al., 2009) [24] | Food insecurity and depression share a bidirectional relationship. | Bidirectional relationship between food insecurity and depression. | Food insecurity and mental health. |
| Results showed that food insecurity and psychological distress had significant bidirectional associations. Findings highlight the mutually reinforcing nature of food insecurity and psychological distress over time, showing that psychological distress was independently predictive of food insecurity. (Kim-Mozeleski et al., 2021) [25] | Food insecurity and psychological distress have bidirectional associations. | Bidirectional relationship between food insecurity and psychological distress. | Food insecurity and mental health. |

**Table S1.** Data analysis of studies on the bidirectional relationship between nutrition and mental health (n=42). *Continued*

| Data units | Summary of data units | Codes (essence of meaning of data units) | Subcategories |
|---|---|---|---|
| The findings from the current study indicate that to adequately address food insecurity, maternal depression should also be addressed and to adequately address maternal depression, food insecurity should also be addressed. (Reesor-Oyer et al., 2021) [26] | Findings support that there is a bidirectional relationship between food insecurity and maternal depression. | Relationship between food insecurity and maternal depression. | Food insecurity and mental health. |
| In total, 34,129 individuals aged ≥50 years [mean (SD) age, 62.4 (16.0) years; 52.1% females] were included in the analysis. Overall, the prevalence of moderate and severe food insecurity was 6.7% and 5.1%, respectively, while the prevalence of depression was 6.0%. Meta-analyses based on country wise estimates showed that overall, moderate food insecurity (vs. no food insecurity) is associated with a nonsignificant 1.69 (95% confidence interval [CI] = 0.82–3.48) times higher odds for depression, while severe food insecurity is significantly associated with 2.43 (95% CI = 1.65–3.57) times higher odds for depression. (Smith et al., 2021) [27] | Results suggest those with severe food insecurity were over two times more likely to suffer from depression (compared with no food insecurity). Utilizing lay health counselors and psychological interventions may be effective mechanisms to reduce depression among food-insecure populations. | Relationship between food insecurity and depression. | Food insecurity and mental health. |
| Random effects meta-analysis was employed to determine the prevalence of food insecurity in SMI and odds ratio (OR) of food insecurity in people with SMI compared to non-psychiatric controls/general population. Twenty-nine unique datasets (31 publications) were included. Prevalence estimate of food insecurity in people with SMI was 40% (95% Ci 29–52%, I2 = 99.7%, N = 27). People with SMI were 2.71 (95% Ci 1.72–3.25) times more likely to report food insecurity than the comparator group (Z = 11.09, p < 0.001, I2 = 95%, N = 23). (Teasdale et al., 2023) [28] | The odds of food insecurity in SMI were higher in high/ high-middle income countries compared to low/low-middle income countries, likely due to the high food insecurity rates in the general population of lower income countries. | Bidirectional relationship between food insecurity and severe mental illnesses. | Food insecurity and mental health. |
| A total of 239 (81.8%) people with SMI and 273 (96.5%) control households were assessed after 12 months. Maintenance of food security or improvement in food insecurity status was observed in 51.5% of households of a person with SMI vs. 39.7% of control households (adjusted risk ratio 1.41: 95% CI 1.11, 1.80). Reduction in symptom severity was indirectly associated with improved FI status via an impact on reducing work impairment and discrimination (P < 0.001). (Tirfessa et al., 2020) [29] | Improving access to mental health care may reduce food insecurity in households of people with SMI. Optimizing engagement in care and adding interventions to improve work functioning and tackle discrimination may further reduce food insecurity. | Relationship between food insecurity and severe mental illnesses. | Food insecurity and mental health. |

**Table S1.** Data analysis of studies on the bidirectional relationship between nutrition and mental health (n=42). *Continued*

| Data units | Summary of data units | Codes (essence of meaning of data units) | Subcategories |
|---|---|---|---|
| According to the findings of this study, there is a significant positive association between depression and obesity in the general population, which appeared to be more marked among women. Further research should focus on underlying factors and examine causal pathways between depression and obesity. (de Wit et al., 2010) [30] | Obesity and depression are associated and more marked among women. | Relationship between obesity and depression. | Obesity and mental health. |
| Regression analyses showed that depressive symptoms at both ages 16 and 18 were associated with increased risk of obesity (BMI≥30) and elevated BMI in young adulthood (age 21) in both black and white girls. Black girls exhibited a significantly greater likelihood of obesity and higher BMI (i.e. a main effect of race), but the race x CES-D interaction was not significant in any analysis. (Franko et al., 2005) [31] | Depressive symptoms in adolescence appear to be predictive of obesity and elevated BMI in early adulthood for both black and white girls, even when taking prior BMI into account, indicating that depressive symptoms confer risk for obesity above and beyond the known tracking of body weight. | Relationship between obesity and depression. | Obesity and mental health. |
| At baseline, 12.9% were overweight, 9.7% were obese, and 8.8% had depressed mood. Baseline depression was not significantly correlated with baseline obesity. Among the 9.7% who were obese at follow-up, 79.6% were obese at baseline, 18.6% were overweight at baseline, and 1.8% were normal weight at baseline. Having depressed mood at baseline independently predicted obesity at follow-up (odds ratio: 2.05; 95% confidence interval: 1.18, 3.56) after controlling for BMI z score at baseline, age, race, gender, parental obesity, number of parents in the home, and family socioeconomic status. (Goodman & Whitaker, 2002) [32] | Depressed adolescents are at increased risk for the development and persistence of obesity during adolescence. Understanding the shared biological and social determinants linking depressed mood and obesity may inform the prevention and treatment of both disorders. | Relationship between obesity and depression. | Obesity and mental health. |
| Among women, depressive symptoms before age 17 years were associated with increased weight gain representing greater risk for adult obesity. Among men, only after controlling for confounders, depressive symptoms before age 17 years were associated with increased weight gain in adulthood but not with occurrence of obesity. (Hasler et al., 2005) [33] | Strong longitudinal association between childhood depressive symptoms and adult BMI. | Relationship between high BMI and depression. | Obesity and mental health. |

**Table S1.** Data analysis of studies on the bidirectional relationship between nutrition and mental health (n=42). *Continued*

| Data units | Summary of data units | Codes (essence of meaning of data units) | Subcategories |
|---|---|---|---|
| A baseline BMI ≥30 significantly increased the odds for subsequent GAD and MDD by 6.27 and 5.25 times, respectively, after adjusting for other significant risk factors. Odds of GAD also increased significantly given a baseline BMI ≥25 (by 2.44 times); however, this association was not independent of other significant risk factors. Predictive associations between a baseline BMI ≥30 and MDD were not attenuated by attained BMI assessed at outcome. (Kasen et al., 2008) [34] | Findings extend existing evidence of the mental health consequences of obesity in a representative sample of mothers and suggest that obesity may have long-term implications for mental distress in women at a clinical level over the adult years. | Relationship between obesity and mental distress. | Obesity and mental health. |
| Elevated depressive symptoms predicted weight gain in men, while changes in depressive symptoms and body weight occurred concurrently in women. Tentative evidence showed that women with excess body weight were more likely to have increased symptoms of depression 10 years later. The findings provide additional support for the reciprocity of influences between depressive symptoms and excess body weight as well as for the importance of gender in modifying these relationships. (Konttinen et al., 2014) [35] | Depressive symptoms may lead to weight gain and weight gain may lead to depressive symptoms, especially in women. | Bidirectional relationship between overweight/obesity and depression. | Obesity and mental health. |
| Results show bidirectional associations between depression and obesity: obese persons had a 55% increased risk of developing depression over time, whereas depressed persons had a 58% increased risk of becoming obese. The association between depression and obesity was stronger than the association between depression and overweight, which reflects a dose-response gradient. (Luppino et al., 2010) [36] | Obesity increased the risk of depression and depression increased the risk of obesity. | Bidirectional relationship between obesity and depression. | Obesity and mental health. |
| BMI was positively associated with the probability of moderate/severe depressive symptoms ($r = 0.49$, $P = 0.03$) and major depression ($r = 0.72$, $P < 0.0001$). The probability curves increased progressively, beginning at BMI of 30. Degree of obesity was an independent risk factor for depression even within the obese population, and women in obesity class 3 (BMI ≥40) were at particular risk (odds ratio (OR) = 4.91, 95% confidence interval (CI): 1.17–20.57), compared to those in obesity class 1 (BMI 30 to <35). (Ma & Xiao, 2010) [37] | Abdominal obesity was positively associated with depressive symptoms, but not major depression, independent of general obesity (BMI). | Relationship between obesity and depression. | Obesity and mental health. |

**Table S1.** Data analysis of studies on the bidirectional relationship between nutrition and mental health (n=42). *Continued*

| Data units | Summary of data units | Codes (essence of meaning of data units) | Subcategories |
|---|---|---|---|
| The results from this large, well-established, long-term cohort study suggest a bidirectional association between depression and obesity in middle-aged and elderly women. (Pan et al., 2012) [38] | Obesity and depression are linked in a bidirectional association. | Bidirectional relationship between obesity and depression. | Obesity and mental health. |
| Participants with childhood major depression had a BMI of 26.1 ± 5.2 as adults, compared with a BMI of 24.2 ± 4.1 in healthy comparisons. This association could not be explained by a number of potentially confounding factors, including age, gender, cigarette or alcohol use, social class, and pregnancy or medication history. (Pine et al., 2001) [39] | Depressed children exhibit a larger BMI as adults than do nondepressed comparisons. This relationship was found in a subsample of participants who did not differ on childhood BMI. This relationship could not be attributed to a number of potential confounding variables. | Relationship between high BMI and depression. | Obesity and mental health. |
| Major depression occurred in 7% of the cohort during early adolescence (11, 13, and 15 years of age) and 27% during late adolescence (18 and 21 years of age). At 26 years of age, 12% of study members were obese. After adjusting for each individual's baseline body mass index (calculated as the weight in kilograms divided by the square of height in meters), depressed late adolescent girls were at a greater than 2-fold increased risk for obesity in adulthood compared with their nondepressed female peers (relative risk, 2.32; 95% confidence interval, 1.29-3.83). (Richardson et al., 2003) [40] | Depression in late adolescence is associated with later obesity, but only among girls. | Relationship between obesity and depression. | Obesity and mental health. |
| Prevalence of moderate or severe depression increased from 6.5% among those with BMI under 25 to 25.9% among those with BMI over 35. Prevalence of obesity increased from 25.4% among those with no depressive symptoms to 57.8% among those with moderate to severe depression. (Simon et al., 2008) [41] | Among middle-aged women, depression is strongly and consistently associated with obesity, lower physical activity and (among the obese) higher caloric intake. | Relationship between obesity and depression. | Obesity and mental health. |

**Table S1.** Data analysis of studies on the bidirectional relationship between nutrition and mental health (n=42). *Continued*

| Data units | Summary of data units | Codes (essence of meaning of data units) | Subcategories |
|---|---|---|---|
| The current analyses supported the hypotheses that the longitudinal relations between depression and obesity are bidirectional and manifold. Among women but not men, depression predicted increased obesity, and obesity predicted increased depression, over a period of 18 years. (Vittengl, 2018) [42] | Depression predicted increased obesity, and obesity predicted increased depression (for women but not men). | Bidirectional relationship between obesity and depression. | Obesity and mental health. |
| After adjusting for potential confounders, participants in the highest tertile of the whole food pattern had lower odds of CES–D depression (OR = 0.74, 95% CI 0.56–0.99) than those in the lowest tertile. In contrast, high consumption of processed food was associated with an increased odds of CES–D depression (OR = 1.58, 95% CI 1.11–2.23). (Akbaraly et al., 2009) [43] | In middle-aged participants, a processed food dietary pattern is a risk factor for CES–D depression 5 years later, whereas a whole food pattern is protective. | Relationship between diet quality and depression. | Diet quality and mental health. |
| Results indicate that psychological distress partially mediated the relationship between diet quality and compulsivity, while diet quality maintained some direct influence on compulsivity having accounted for distress. Compulsivity is a transdiagnostic phenotype cutting across a range of mental illnesses including obsessive-compulsive disorder, substance related and addictive disorders. (Brierley et al., 2021) [44] | Diet quality may influence mental illnesses (including obsessive-compulsive disorder, substance related and addictive disorders), and mental illnesses predict diet quality. | Bidirectional relationship between diet quality and mental illnesses. | Diet quality and mental health. |
| Our present study innovatively extended existing evidence demonstrating bidirectional associations between the Dietary Diversity Scale and depressive symptoms simultaneously in the same longitudinal cohort by a cross lagged model. The demonstrated associations suggested that maintaining high dietary diversity can reduce the incidence of depressive symptoms, and increased depressive symptoms are not conducive to maintaining high dietary diversity. (Dong et al., 2024) [45] | Diversity in the diet (involving eating foods from different food groups) reduces depression and low diet diversity is associated with depression. | Bidirectional relationship between diet diversity and depression. | Diet quality and mental health. |

**Table S1.** Data analysis of studies on the bidirectional relationship between nutrition and mental health (n=42). *Continued*

| Data units | Summary of data units | Codes (essence of meaning of data units) | Subcategories |
|---|---|---|---|
| After adjustments for age, socioeconomic status, education, and health behaviors, a "traditional" dietary pattern characterized by vegetables, fruit, meat, fish, and whole grains was associated with low er odds for major depression or dysthymia and for anxiety disorders. A "western" diet of processed or fried foods, refined grains, sugary products, and beer was associated with a higher GHQ -12 score. There was also an inverse association between diet quality score and GHQ 12 score that was not confounded by age, socioeconomic status, education, or other health behaviors. (Jacka et al., 2010) [46] | These results demonstrate an association between habitual diet quality and the high-prevalence mental disorders, although reverse causality and confounding cannot be ruled out as explanations. Further prospective studies are warranted. | Relationship between diet quality and mental disorders. | Diet quality and mental health. |
| A priori healthy diet quality score was inversely related to depression (odds ratio [OR] = 0.71, 95% confidence interval [CI] = 0.59-0.84) and anxiety (OR = 0.77, 95% CI = 0.68-0.87) in women and to depression (OR = 0.83, 95% CI = 0.70-0.99) in men. (Jacka et al, 2011) [47] | In this study, those with better quality diets were less likely to be depressed, whereas a higher intake of processed and unhealthy foods was associated with increased anxiety. | Relationship between diet quality and depression/anxiety. | Diet quality and mental health. |
| There were cross-sectional, dose response relationships identified between measures of both healthy (positive) and unhealthy (inverse) diets and scores on the emotional subscale of the Pediatric Quality of Life Inventory (PedsQL), where higher scores mean better mental health, before and after adjustments for age, gender, socio-economic status, dieting behaviours, body mass index and physical activity. Higher healthy diet scores at baseline also predicted higher PedsQL scores at follow-up, while higher unhealthy diet scores at baseline predicted lower PedsQL scores at follow-up. (Jacka et al, 2011) [48] | Improvements in diet quality were mirrored by improvements in mental health over the follow-up period, while deteriorating diet quality was associated with poorer psychological functioning. | Relationship between diet quality and psychological functioning. | Diet quality and mental health. |
| Both linear and logistic regression analyses were used to examine whether or not diet quality was associated with depressive symptoms. The dependent variable was depressive symptoms and independent variables included HEI-2005, race, sex, age, education, income, and food-assistance program participation. Mean HEI-2005 score was 52.17±0.40 (out of 100). Mean Center for Epidemiologic Studies Depression scale score was 11.64±0.25 (out of 40). (Kuczmarski et al., 2010) [49] | Diet quality was significantly associated with reported symptoms of depression. However, income was a significantly stronger predictor of depression compared to diet quality, education, and sex. | Relationship between diet quality and depression. | Diet quality and mental health. |

**Table S1.** Data analysis of studies on the bidirectional relationship between nutrition and mental health (n=42). *Continued*

| Data units | Summary of data units | Codes (essence of meaning of data units) | Subcategories |
|---|---|---|---|
| The healthy diet pattern was significantly associated with a reduced odds of depression (OR: 0.84; 95% CI: 0.76, 0.92; P , 0.001). No statistically significant association was observed between the Western diet and depression (OR: 1.17; 95% CI: 0.97, 1.68; P = 0.094); however, the studies were too few for a precise estimate of this effect. (Lai et al., 2014) [50] | The results suggest that high intakes of fruit, vegetables, fish, and whole grains may be associated with a reduced depression risk. | Relationship between diet quality and depression. | Diet quality and mental health. |
| A healthy Japanese dietary pattern characterized by high intakes of vegetables, fruit, mushrooms and soy products was associated with fewer depressive symptoms. The multivariate-adjusted odds ratios (95% confidence intervals) of having depressive symptoms for the lowest through highest tertiles of the healthy Japanese dietary pattern score were 1.00 (reference), 0.99 (0.62–1.59) and 0.44 (0.25–0.78), respectively (P for trend ¼ 0.006). Other dietary patterns were not appreciably associated with depressive symptoms. (Nanri et al., 2010) [51] | Findings suggest that a healthy Japanese dietary pattern may be related to decreased prevalence of depressive status. | Relationship between diet quality and depression. | Diet quality and mental health. |
| After a median follow-up of 4.4 years, 480 new cases of depression were identified. The multiple adjusted hazard ratios (95% confidence intervals) of depression for the 4 upper successive categories of adherence to the MDP (taking the category of lowest adherence as reference) were 0.74 (0.57-0.98), 0.66 (0.50-0.86), 0.49 (0.36-0.67), and 0.58 (0.44-0.77) (P for trend <.001). Inverse dose-response relationships were found for fruit and nuts, the monounsaturated- to saturated-fatty acids ratio, and legumes. (Sánchez-Villegas et al., 2009) [52] | Results suggest a potential protective role of the MDP with regard to the prevention of depressive disorders; additional longitudinal studies and trials are needed to confirm these findings. | Relationship between diet quality and depression. | Diet quality and mental health. |
| A higher risk of depression was associated with consumption of fast food. No linear relationship was found between the consumption of commercial baked goods and depression. Participants belonging to consumption quintiles Q2–Q5 showed an increased risk of depression compared with those belonging to the lowest level of consumption. (Sánchez-Villegas et al., 2012) [53] | Fast-food and commercial baked goods consumption may have a detrimental effect on depression risk. | Relationship between diet quality and depression. | Diet quality and mental health. |

**Table S1.** Data analysis of studies on the bidirectional relationship between nutrition and mental health (n=42). *Continued*

| Data units | Summary of data units | Codes (essence of meaning of data units) | Subcategories |
|---|---|---|---|
| Although the taking of the probiotic did not generally change the mood, this appeared to be a reflection of the generally good mood in this sample. When those in the bottom third of the depressed/elated dimension at baseline were considered, they selectively responded by reporting themselves as happy rather than depressed after taking the probiotic. (Benton et al., 2007) [54] | The consumption of a probiotic-containing yoghurt improved the mood of those whose mood was initially poor. | Relationship between probiotic (through gut microbiome) and mood/depression. | Diet/gut and mental health. |
| Alterations in central GABA receptor expression are implicated in the pathogenesis of anxiety and depression, which are highly comorbid with functional bowel disorders. In this work, we show that chronic treatment with L. rhamnosus induced region-dependent alterations in the brain with increases in cortical regions and concomitant reductions in expression in the hippocampus, amygdala, and locus coeruleus, in comparison with control-fed mice. (Bravo et al., 2011) [55] | Findings highlight the important role of bacteria in the bidirectional communication of the gut–brain axis and suggest that certain organisms may prove to be useful therapeutic adjuncts in stress related disorders such as anxiety and depression. | Relationship between probiotic (through gut microbiome) and anxiety and depression. | Diet/gut and mental health. |
| In stress-sensitive rats, absence of the gut microbiota exacerbates the neuroendocrine and behavioral responses to acute stress and the results coexist with alterations of the dopaminergic turnover rate in brain upper structures that are known to regulate reactivity to stress and anxiety-like behavior. (Crumeyrolle-Arias et al., 2014) [56] | These findings are further proofs of the crucial influence of the gut microbiota on several aspects of brain function. Thus it is plausible that gut microbiota dysbiosis that can occur at various life stages may contribute to the development of neuropsychiatric disorders. | Relationship between gut microbiota and neuropsychiatric disorders. | Diet/gut and mental health. |
| In the preclinical study, rats were daily administered PF for 2 weeks and subsequently tested in the conditioned defensive burying test, a screening model for anti-anxiety agents. In the clinical trial, volunteers participated in a double-blind, placebo-controlled, randomised parallel group study with PF administered for 30 d and assessed with the Hopkins Symptom Checklist, the Hospital Anxiety and Depression Scale, the Perceived Stress Scale, the Coping Checklist and 24 h urinary free cortisol. (Messaoudi et al., 2011) [57] | Daily subchronic administration of PF significantly reduced anxiety-like behavior in rats (P,0·05) and alleviated psychological distress in volunteers. | Relationship between probiotics (through gut microbiome) and anxiety/psychological distress. | Diet/gut and mental health. |

**Table S1.** Data analysis of studies on the bidirectional relationship between nutrition and mental health (n=42). *Continued*

| Data units | Summary of data units | Codes (essence of meaning of data units) | Subcategories |
|---|---|---|---|
| Results found a significant rise in both Lactobacillus and Bifidobacteria in those taking the LcS, and there was also a significant decrease in anxiety symptoms among those taking the probiotic vs controls ($p = 0.01$). (Rao et al., 2009) [58] | These results lend further support to the presence of a gut-brain interface, one that may be mediated by microbes that reside or pass through the intestinal tract. | Relationship between probiotics (through gut microbiome) and anxiety. | Diet/gut and mental health. |
| Fecal microbiota transplantation of germ free mice with 'depression microbiota' derived from MDD patients resulted in depression-like behaviors compared with colonization with 'healthy microbiota' derived from healthy control individuals. Mice harboring 'depression microbiota' primarily exhibited disturbances of microbial genes and host metabolites involved in carbohydrate and amino acid metabolism. (Zheng et al., 2016) [59] | This study demonstrates that dysbiosis of the gut microbiome may have a causal role in the development of depressive-like behaviors, in a pathway that is mediated through the host's metabolism. | Relationship between gut microbiome and depression. | Diet/gut and mental health. |

*Notes*. BMI = body mass index; CES–D = center for epidemiologic studies depression scale; CI = confidence intervale; DRI = dietary reference intakes; FI = food insecurity; GABA = gamma-aminobutyric acid; GAD = generalized anxiety disorder; GHQ-12 = 12-item general health questionnaire; HEI = healthy eating index; LcS = lactobacillus casei strain Shirota; MDD = major depressive disorder; MDP = mediterranean dietary pattern; OR = odds ratio; PedsQL = pediatric quality of life inventory; PF = probiotic formulation; SMI = severe mental illness; SD = standard deviation
